# Hnf4α is a key gene that can generate columnar metaplasia in oesophageal epithelium

**DOI:** 10.1016/j.diff.2016.11.001

**Published:** 2017

**Authors:** Benjamin J. Colleypriest, Zoë D. Burke, Leonard P. Griffiths, Yu Chen, Wei-Yuan Yu, Ramiro Jover, Michael Bock, Leigh Biddlestone, Jonathan M. Quinlan, Stephen G. Ward, J. Mark Farrant, Jonathan M.W. Slack, David. Tosh

**Affiliations:** a Centre for Regenerative Medicine, Department of Biology and Biochemistry, University of Bath, Claverton Down, Bath BA2 7AY, UK; b Department of Gastroenterology, Royal United Hospital, Combe Park, Bath BA1 3NG, UK; c Unidad Mixta Hepatologia Experimental & CIBERehd, Departamento de Bioquimica y Biologia Molecular, Universidad de Valencia, Spain; d Department of Gastroenterology, Hepatology and Endocrinology, Hannover Medical School, Hannover, Germany; e Department of Pharmacy and Pharmacology, University of Bath, Claverton Down, Bath BA2 7AY, UK; f Stem Cell Institute, University of Minnesota, Minneapolis 55455, USA

**Keywords:** Barrett's oesophagus, HNF4α, Hepatocyte nuclear factor 4-alpha, Oesophageal cancer, Metaplasia

## Abstract

Barrett's metaplasia is the only known morphological precursor to oesophageal adenocarcinoma and is characterized by replacement of stratified squamous epithelium by columnar epithelium. The cell of origin is uncertain and the molecular mechanisms responsible for the change in cellular phenotype are poorly understood. We therefore explored the role of two transcription factors, Cdx2 and HNF4α in the conversion using primary organ cultures. Biopsy samples from cases of human Barrett's metaplasia were analysed for the presence of CDX2 and HNF4α. A new organ culture system for adult murine oesophagus is described. Using this, *Cdx2* and *HNF4α* were ectopically expressed by adenoviral infection. The phenotype following infection was determined by a combination of PCR, immunohistochemical and morphological analyses. We demonstrate the expression of CDX2 and HNF4α in human biopsy samples. Our oesophageal organ culture system expressed markers characteristic of the normal SSQE: p63, K14, K4 and loricrin. Ectopic expression of *HNF4α,* but not of *Cdx2* induced expression of *Tff3*, *villin,* K8 and E-cadherin. *HNF4α* is sufficient to induce a columnar-like phenotype in adult mouse oesophageal epithelium and is present in the human condition. These data suggest that induction of *HNF4α* is a key early step in the formation of Barrett's metaplasia and are consistent with an origin of Barrett's metaplasia from the oesophageal epithelium.

## 1 Introduction

Barrett's metaplasia (BM) is a pathological condition characterized by replacement of stratified squamous epithelium (SSQE) of the distal oesophagus by columnar epithelium (Fitzgerald, 2006, Spechler and Goyal, 1996). BM is found in the context of gastro-oesophageal reflux disease (GORD) and arises as a consequence of the damage provoked by acid and bile (Vaezi and Richter, 1996, Falk, 2002). The condition is important because it is the only known morphological precursor to oesophageal adenocarcinoma (OA). OA has a poor prognosis with a five year survival of between 5% and 15% (Nur et al., 2013). The incidence of OA has increased dramatically in the western world over the last 30 years, at a faster rate than any other cancer (Pohl and Welch, 2005, Bollschweiler et al., 2001). Despite considerable research, the molecular mechanisms responsible for the induction of columnar epithelium, and the precise cellular origin of BM, remain unknown (Fitzgerald, 2006, Souza et al., 2008, Spechler et al., 2010, Chen et al., 2011, Quinlan et al., 2007). Plausible candidates for the cell of origin, are the oesophageal epithelium itself, the oesophageal glands, or multipotent cells residing near the oesophageal-gastric junction (Coad et al., 2005, Leedham et al., 2008, Wang et al., 2011, Nicholson et al., 2012, Streppel et al., 2014, Clemons et al., 2014).

Several lines of evidence suggest that the caudal related homeobox genes (*CDX*) *1* and *2* are involved in the initiation of BM (Souza et al., 2008). CDX1 and CDX2 are important transcription factors in the regional patterning of the caudal gut during embryonic development, and in the differentiation of the intestinal epithelium (Gao et al., 2009, Silberg et al., 2000). The expression pattern of both genes is restricted to the endodermal epithelium that is destined to become the small and large intestine (Silberg et al., 2000). Ectopic expression of *Cdx2* in the stomach of transgenic mice can cause the formation of heterotopic intestinal epithelium (Mutoh et al., 2004, Silberg et al., 2002). Conversely, selective deletion of gut endodermal *Cdx2* during development results in the expression of squamous differentiation markers in the intestine (Gao et al., 2009). Mice heterozygous for a null allele of *Cdx2* develop patches of SSQE reminiscent of oesophageal epithelium within the colon and small intestine (Chawengsaksophak et al., 1997). Cdx1 and 2 also control the rostral-caudal pattern of tissue types and body parts: for example, loss of Cdx2 function results in an anterior homeotic shift in vertebrae (Van Den Akker et al., 2002) and intestine (Gao et al., 2009, Chawengsaksophak et al., 1997).

Both *CDX1* and *CDX2* are aberrantly expressed in BM and in adjacent squamous epithelium (Eda et al., 2003, Silberg et al., 1997). Since the oesophagus is exposed to acid and bile during GORD, this suggests a potential mechanism of action for the initiation of BM (Marchetti et al., 2003, Kazumori et al., 2006). Exposure to acid and bile has been shown to induce expression of *Cdx1* and *Cdx2* in oesophageal cells in rats (Kazumori et al., 2006). Given the potential role of *CDX2* in the development of BM, we wished to determine whether ectopic *Cdx2* expression was able to induce a columnar-like phenotype in murine oesophageal cultures.

We were also interested to know whether other transcription factors might be involved in the conversion of SSQE to columnar epithelium. Hepatocyte nuclear factor 4α (HNF4α), a nuclear receptor type transcription factor, may also be considered as a candidate for the initiation of BM. During early development of the gut, *Hnf4α* is expressed in the intestine as well as the stomach, kidney, liver and pancreas (Zhong et al., 1993, Taraviras et al., 1994). Importantly, *HNF4α* is not expressed in normal human oesophagus, but is expressed in BM (Piessen et al., 2007, Green et al., 2014, Wang et al., 2009). Normal epithelial differentiation of the colon and maturation of goblet cells is dependent upon the presence of Hnf4α (Garrison et al., 2006). Therefore, we wanted to know whether ectopic expression of Cdx2 and/or Hnf4α might incite Barrett's-like changes in squamous cells.

To address the potential role of *CDX2* and *HNF4α* in BM we developed a long-term adult mouse oesophageal explant model. Long-term culture of oesophageal epithelium has proven difficult. Broadly there are two sources of cells available to study BM: immortalized cell lines such as Het-1A, or *ex vivo* primary cell culture involving either mechanical tissue mincing or enzymatic digestion of oesophageal tissue. Cells or explants have been cultured on a variety of substrates, matrices and scaffolding, including organotypic models with multi-layered squamous cells (Green et al., 2010). While each of these models has advantages and disadvantages, for our purposes we ideally needed the following features: first, the full repertoire of squamous cells expressing basal cell markers (cytokeratin 14 (K14) and p63), differentiating markers (K4 and involucrin) and a terminally differentiated cell marker (loricrin); second a feeder-free model to simplify characterisation and experimental interpretation; and third cell viability for at least two weeks to allow for gene insertion. None of the existing models satisfied all these criteria. We developed and characterized our oesophageal model, transduced oesophageal explants with adenoviral vectors expressing *Cdx2* or *HNF4α* and analysed the phenotype of the cells.

Our study directly investigated the ability of *Cdx2* and *HNF4α* overexpression to induce an intestinal columnar phenotype in a model of adult oesophageal epithelium. Contrary to previous expectations, but consistent with some other recent studies (Kong et al., 2011, Kong et al., 2009), introduction of *Cdx2* did not provoke a columnar phenotype with expression of intestinal genes. However, we found that *HNF4α* did so. Since we also confirm that *HNF4α* is expressed in BM, we consider that its ectopic activation is likely to be a key early step in the formation of BM. The fact that the changes are provoked in cultures of normal oesophageal epithelium are consistent with the possibility that BM does arise from the oesophageal epithelium, although cannot exclude the other possibilities.

## 2 Result

### 2.1 Expression of Cdx2 and HNF4α along the normal GI tract and in Barrett's metaplasia

**Fig. 1:**
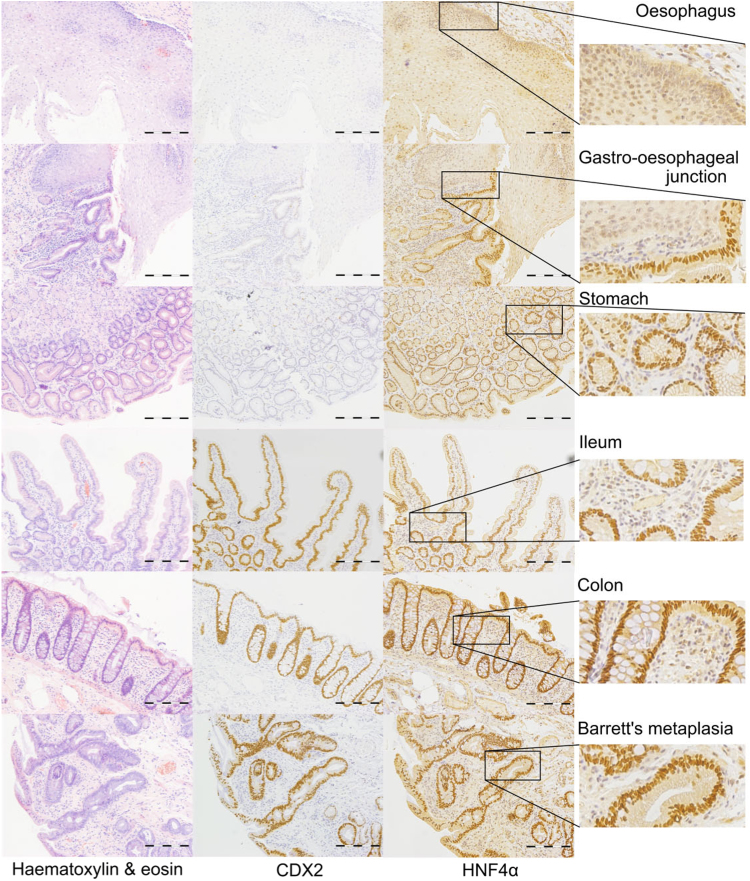
Expression of Cdx2 and HNF4α in Barrett's metaplasia Immunohistochemical staining for CDX2 and HNF4α (brown) in sections of normal oesophagus, gastro-oesophageal junction, stomach, ileum, colon and Barrett's metaplasia. Sections counterstained with Gill's haematoxylin. H&E staining of similar sections are also shown. Staining for HNF4α in oesophagus represents background staining. Scale bar represents 200 µm.

Previous studies have described the expression of CDX2 in Barrett's epithelium but the involvement of HNF4α is less well documented. Here we demonstrate using immunohistochemical analysis of normal human oesophagus and Barrett's epithelium that HNF4α protein is indeed present in BM in an identical pattern to that of CDX2 (Fig. 1). HNF4α background staining was not eliminated from the slide sections of oesophagus, but contrast is demonstrated at the gastro-oesophageal section.

### 2.2 Oesophageal explants are viable *in vitro* for up to 3 months

**Fig. 2:**
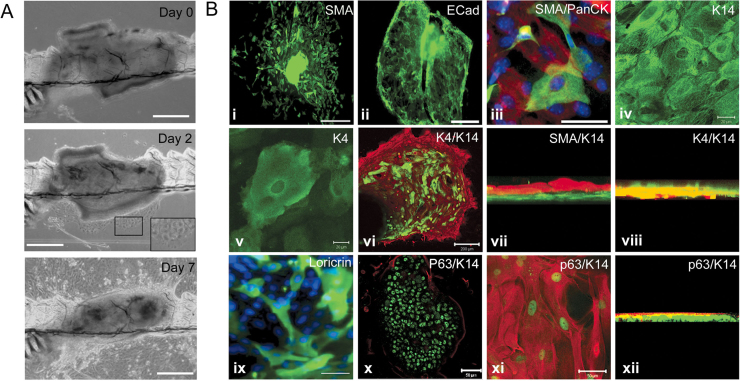
Characterisation of oesophageal explant cultures. (A) Brightfield images of a single mouse oesophageal explant followed over 7 days of culture. An outgrowth of cells is first observed after 2 days of culture (see inset for higher magnification), and increases in size thereafter. Scale bars represent 250 µm. (B) Immunofluorescent staining of oesophageal explants for (i) SMA, (ii) E-cad, (iii) SMA/PanCK (red/green), (iv) K14, (v) K4, (vi) K4/K14 (green/red), (vii) SMA/K14 (green/red), (viii) K4/K14 (green/red), (ix) Loricrin and (*x*-xii) p63/K14 (green/red). Z-stack images are shown for clarity (vii, viii and xii). Scale bars represent 500 µm (i/ii), 200 µm (vi), 100 µm (ix), 50 µm (iii/x/xi) and 20 µm (iv/v). (For interpretation of the references to color in this figure legend, the reader is referred to the web version of this article.)

We have developed a new culture system for adult mouse oesophagus, to complement the system previously developed for embryonic oesophagus. (Yu et al., 2005). Adult mouse oesophageal explants attached to plastic substratum within 48 h of plating in 82% of cases (41/50). In 95% (39/41) of explants that attached, cells migrated out from the explants within a week (Fig. 2). Two distinct cell morphologies were found around each explant: mesenchymal and epithelial. A central area surrounding the original explant exhibited overlapping cells, comprising a multilayered structure. The size of the outgrowth increased daily for 2–3 weeks before reaching equilibrium and remained viable for up to three months (Fig. 2A; Supplementary Fig 1).

### 2.3 Characterisation of oesophageal explant cultures

To assess the adult oesophageal explant culture as an *in vitro* model of squamous oesophagus, cellular phenotypes were characterized by immunofluorescence detection using proteins typically found in the native oesophageal structure. The outgrowth of tissue surrounding the oesophageal explant contained mesenchymal cells expressing smooth muscle actin (SMA, Fig. 2Bi, iii and vii) and E-cadherin-positive epithelial cells (Fig. 2Bii). The majority of outgrowths (36/39; >90%) contained both epithelial and mesenchymal cell types. A minority of explants contained only SMA-positive cells, but no outgrowth consisted purely of epithelium, suggesting that mesenchymal cells are required for the maintenance of the epithelial cells.

**Fig. 3:**
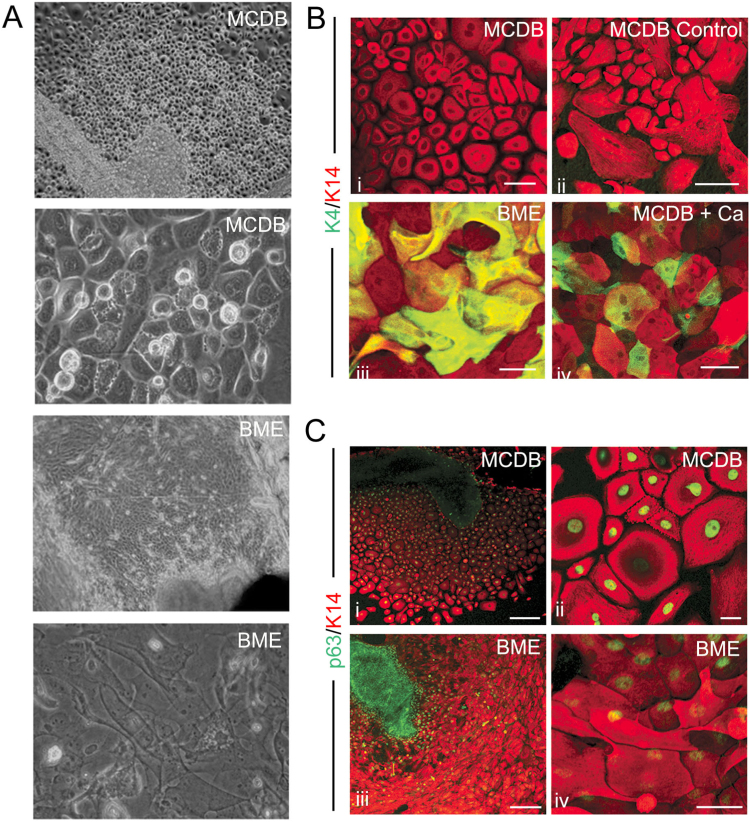
Calcium provokes stratification of oesophageal epithelium. (A) Brightfield images showing oesophageal explant morphology in MCDB 153 medium (calcium concentration 0.03 mM) and BME medium (calcium concentration 1.8 mM). (B) Immunofluorescent staining for K4 and K14 in oesophageal explants cultured in MCDB 153 (i) or BME (iii) for 7 days. Also shown are explants cultured for 5 days in MCDB followed by 3 days culture in 1 mM calcium (iv – compare to 8 days culture in MCDB ii). (C) Immunofluorescent staining for p63 and K14 in oesophageal explants cultured in MCDB 153 (i and ii) or BME (iii and iv). Scale bars represent 20 µm (Cii) 50 µm (B i, B ii and C iv), 100 µm (B ii and B iv) and 200 µm (C I and C ii).

Three different markers of squamous differentiation were examined within the explant cultures: K14 (basal)-, K4 (suprabasal)- and loricrin (the major component of the cornified cell envelope)- expressing cells were all present in the cultures (Fig. 2B iv-xii). K4-positive cells were found above the K14 layer (Fig. 2Bvi and viii). The transcription factor p63, required for the induction and maintenance of the oesophageal SSQE (Daniely et al., 2004), was expressed in two distinct patterns (Fig. 2Bx-xii). The first type of p63-positive cell was found within or immediately adjacent to the explant and lacked K14 expression (Fig. 2Bx and Fig. 3Ciii). The second was in cells co-expressing p63 with K14 and were more commonly located in the area surrounding the explant (Fig. 2Bxi and Fig. 3Ciii). The epithelium surrounding these cells was positive for K14 but negative for p63 (Fig. 3Ciii). The K14/p63 co-expressing cells were covered by a layer of K14-positive cells (Fig. 2Bxii).

### 2.4 Role of calcium in oesophageal differentiation

Calcium is an essential determinant of epidermal keratinocyte proliferation and differentiation (Hennings and Holbrook, 1983, Hennings et al., 1980). We wished to determine whether SSQE behaves in a similar fashion to skin in its response to calcium. To address this we cultured oesophageal explants in either BME or MDCB 153 medium which contain 1.8 mM (normal) and 0.03 mM (low) calcium respectively (Fig. 3). Explants cultured in normal calcium had a different morphology to those cultured in low calcium (Fig. 3A). Oesophageal cells cultured for seven days in low calcium grew as monolayers, failed to form cell-cell contacts, and did not stratify, as judged by the absence of K4 (Fig. 3Bi). These conditions facilitated quantification of staining and greatly improved viral infection efficiency (see below) In contrast, cells cultured in BME showed robust staining for K4 (Fig. 3Biii *vs* Bi). To test the response to calcium, we cultured oesophageal explants for 5 days in 0.03 mM calcium followed by 3 days at a final concentration of 1 mM calcium (Fig. 3Biv). In low calcium conditions cells did not express K4 (Fig. 3Bii) but after short term exposure to higher concentrations of calcium, K4 became expressed in approximately 25% of the cells (Fig. 3Biv). Expression of p63 was maintained in most (99%) of the cells cultured in low calcium and was co-expressed exclusively with K14. This contrasts with oesophageal explants cultured in normal calcium, where cells expressing K14 alone can be found (Fig. 3C compare ii and iv).

### 2.5 Cdx2 represses p63 expression but does not induce intestinal genes

**Fig. 4:**
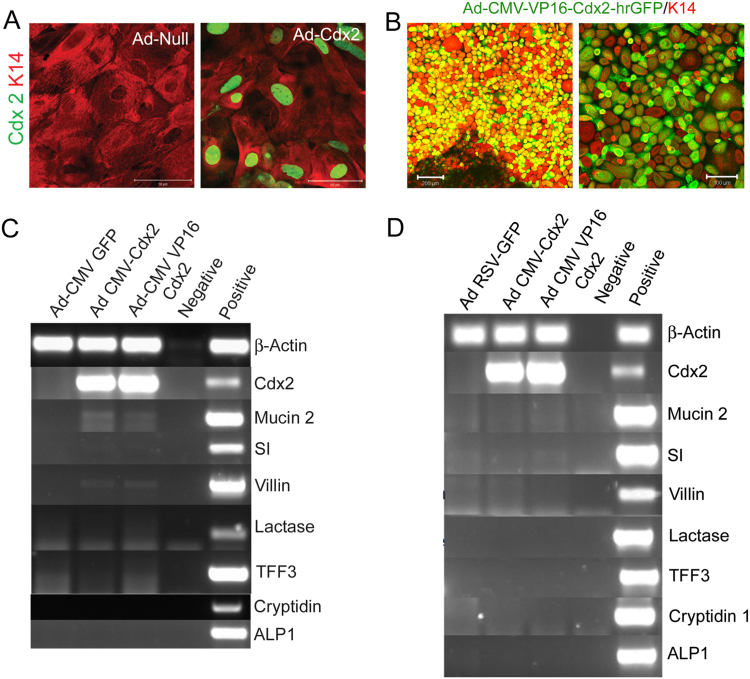
Ectopic expression of Cdx2 in oesophageal explants does not induce a columnar phenotype. Immunofluorescent staining for K14 (red) and Cdx2 (surrogate green from GFP) in Ad-null or Ad-CMV-Cdx2-hrGFP infected oesophageal explants cultured in MCDB 153 (A) or BME media (B) RT-PCR analysis for: *β-actin, Cdx2, Mucin 2, SI, villin, lactase, Tff3, cryptidin* and *alkaline phosphatase* in Ad-RSV-GFP, Ad-CMV-Cdx2-hrGFP or Ad-CMV-VP16Cdx2-hrGFP virus infected oesophageal explants cultured in MCDB 153 (C) or BME media (D). Scale bars represent 200 µm and 100 µm (A) and 50 µm (B). (For interpretation of the references to color in this figure legend, the reader is referred to the web version of this article.)

We examined the efficacy of the *Ad-CMV-Cdx2-eGFP* virus in adult oesophageal explants, cultured in low (MCDB 153) and normal (BME) calcium concentrations, to induce intestinal gene expression (Fig. 4). Explants were grown for 7 days, incubated with medium containing virus for 12 h, and the expression of intestinal markers was assessed by immunofluorescence and RT-PCR after 3 further days of culture. The presence of Cdx2 protein within the nuclei of K14-positive epithelial cells was determined by GFP expression (Fig. 4A and B) and *Cdx2* mRNA was detected by RT-PCR (Fig. 4C and D). In cells cultured in normal calcium, incubation with *Ad-CMV-Cdx2-eGFP* resulted in robust expression of *Cdx2* within the majority of epithelial cells (Fig. 4A) with a transfection efficiency of 73%. In low calcium cultures, we initially used the same titre of virus as for the BME cultures but as this resulted in significant cell death the titre was reduced 100-fold to 5×10^5^ IU per explant to maintain viability and produce a similar percentage of GFP-positive cells (>50%) (Fig. 4B) as for the BME cultures. Three days following *Cdx2* infection, p63 was lost from some of the cells expressing Cdx2 but not from cells infected with control adenovirus (4.33+/−1.53 cells per high power field lost p63 expression compared with 0.33+/- 0.58; p=0.046 Mann-Whitney test) (Supplementary Fig 2). It was only possible to determine the p63 loss because of the monolayer morphology and the fact that the vast majority of cells within the low calcium culture were p63-positive.

Despite the high level of expression of *Cdx2* in the low calcium medium, the levels of induction of *Mucin2* and *Villin* RNA were only just detectable (Fig. 4C – 35 cycles of PCR) and were not visible at the protein level. Ectopic expression of *Cdx2* did not induce detectable expression of the intestinal markers *Mucin 2, Sucrase isomaltase (SI)*, *Villin*, *Lactase*, *Trefoil factor 3 (Tff3)*, *Alkaline phosphatase 1(ALP1)*, or *Cryptdin 1* (Fig. 4D) in BME. This confirms the limited effect of *Cdx2* overexpression in driving authentic oesophageal epithelial cells to an intestinal columnar phenotype.

### 2.6 HNF4α induces a columnar-like phenotype in oesophageal explant cultures

**Fig. 5:**
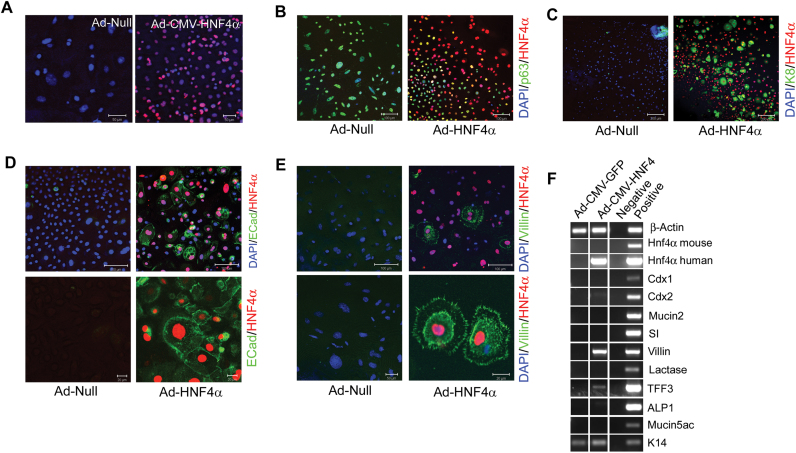
HNF4α transduction induces a columnar-like phenotype in oesophageal explants. Immunofluorescent staining for HNF4α (A), p63/HNF4α (B), K8/HNF4α (C), Ecad/HNF4α (D) and Villin/HNF4α in Ad-null or Ad-CMV-HNF4α infected oesophageal explants cultured in MCDB 153 medium. DAPI counterstain is also shown (A). RT-PCR analysis for *β-actin, Cdx2* (mouse and human)*, Mucin 2, SI, villin, lactase, Tff3, alkaline phosphatase 1, Mucin 5ac and K14* in Ad-null or Ad-CMV-HNF4α infected oesophageal explants cultured in MCDB 153 medium. Scale bars are as indicated.

We tested the effects of ectopic expression of *HNF4α* on oesophageal explants cultured in low calcium medium. A transfection efficiency of 96% was achieved with expression of human HNF4α protein was confirmed by immunofluorescence (Fig. 5A). Co-staining for HNF4α and p63 in control and *HNF4α* infected cultures revealed a reduction in the number of p63-positive cells from 98% in HNF4α-infected cultures to 32% in control infected cultures (n=3; S.D +/- 11.6%) indicating that HNF4α suppresses the SSQE phenotype (Supplementary Fig 3).

In addition we examined expression of the columnar marker cytokeratin 8 (K8) (Yu et al., 2005), E-cadherin and villin (Fig. 5C-E respectively). We found that E-cadherin is not expressed in the low calcium cultures but that it becomes robustly expressed in the presence of HNF4α (Fig. 5D). HNF4α also induces expression of cytokeratin 8 (K8) and villin (Fig. 5C and E). To ascertain whether HNF4α was able to provoke an intestinal columnar phenotype, we determined the expression of *Cdx1*, *Cdx2*, *Mucin2*, *SI*, *Villin*, *Lactase*, *Tff3*, *ALP1,* the stomach mucin *Muc5AC* and *K14* by PCR. *Villin* was robustly expressed following ectopic *HNF4α* expression, while expression of the transcription factor *Tff3* was induced to a lesser extent. All other mRNAs examined were not detected (Fig. 5F).

We also tested the effects of Cdx2 and HNF4α in combination but conditions could not be found in which the cultures remained viable.

### 2.7 Adenoviral expression of Cdx2, HNF1α and HNF4α in Het-1A cells

**Fig. 6:**
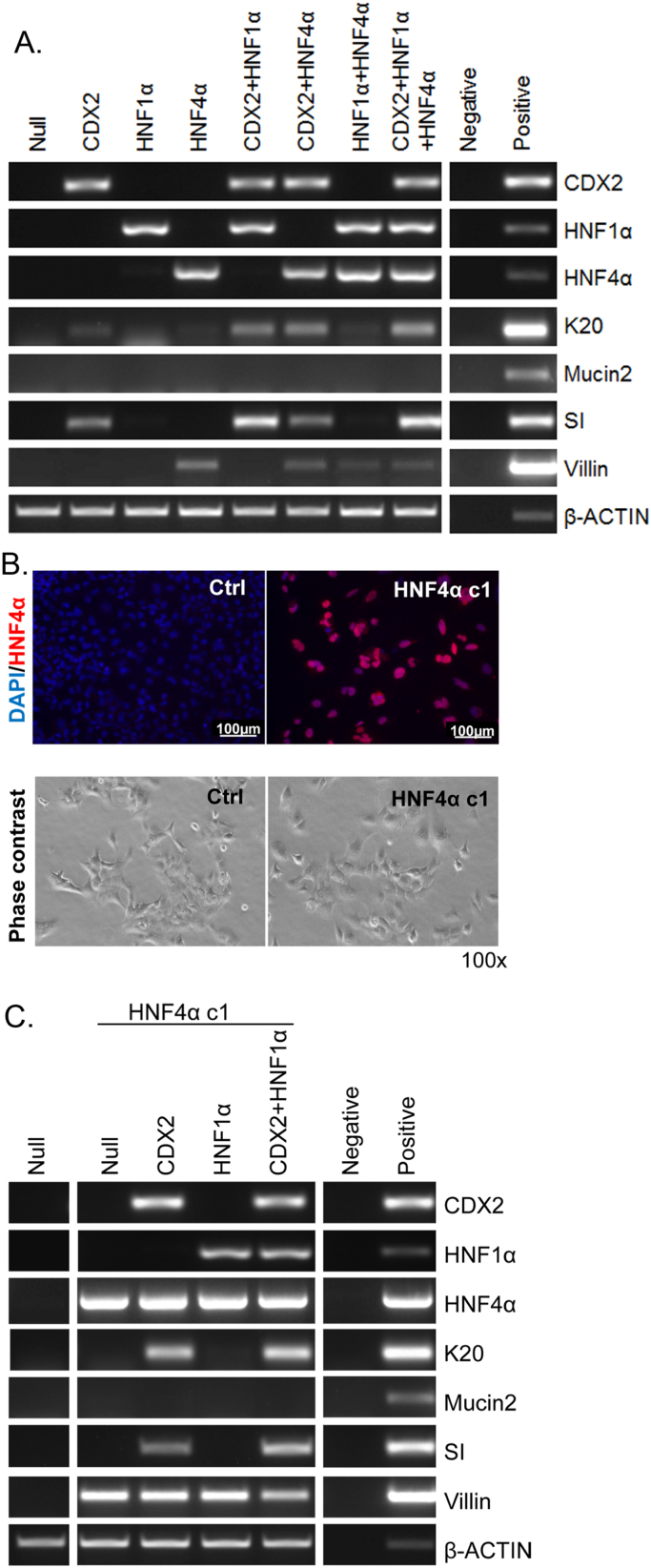
Expression of HNF4α and Cdx2 in Het-1A cells induces the expression of intestinal genes. (A) RT-PCR analysis for *Cdx2*, *HNF1α*, *HNF4α*, *K20*, *Mucin2*, *SI*, *Villin* and *β-actin* in Het-1A cells transiently infected with Ad-Null, Ad-Cdx2, Ad-HNF4α, Ad-HNF1α virus alone or in combination as indicated. (B) Immunofluorescent staining for HNF4α and phase contrast images of the stable HNF4α-expressing Het-1A clone Hnf4α-c1. (C) RT-PCR analysis (as above) of stable Hnf4α-c1 cells infected with Ad-Null, Ad-Cdx2, Ad-HNF1α alone or in combination as indicated.

Because it was not practicable to test the combined effect of Cdx2 and HNF4α on the oesophageal explant model, for this purpose we used the human Het-1A oesophageal cell line. In these experiments an additional gene, *HNF1α*, was included. *HNF1α* plays a crucial role in intestinal development so we wished to determine if co-expression with *HNF4α* and *Cdx2* could further enhance intestinal gene expression. Het1A cells were infected with virus encoding *Cdx2*, *HNF4α* and *HNF1α* alone or in combination and analysed by RT-PCR for the induction of *Mucin2*, *K20*, *SI* and *Villin* (Fig. 6A). Infection with *HNF4α* alone induced expression of villin, while *Cdx2* provoked the expression of *K20* and *SI*. Combined infection with *HNF4α* and *Cdx2* resulted in the induction of *villin*, *K20* and *SI.* Infection with *HNF1α* enhanced *Cdx2* induced expression of *K20* and *SI* but had a negligible effect on its own. Interestingly, *HNF1α* expression appeared to have an antagonistic effect on *HNF4α* mediated induction of *villin*.

We also generated stable HNF4α-expressing Het-1A cells (Het-1A-HNF4α c1). Expression of HNF4α protein in the Het-1A-HNF4α c1 clone was confirmed by immunofluorescence (Fig. 6B) and induction of intestinal gene expression analysed by RT-PCR (Fig. 6C). Robust expression of *Villin* was induced in the stable Het-1A-HNF4α c1 clone. Subsequent infection with *Cdx2* induced *K20* and *SI* expression*,* while *HNF1α* did not. *K20* and *SI* were induced following combined infection with *Cdx2* and *HNF1α* whereas *villin* expression was reduced. Quantitative RT-PCR analysis revealed a significant increase in *villin* expression in *HNF4α* transiently transfected Het-1A cells and the stable Het-1A-HNF4α c1 clone (compared to uninfected controls) but this was not significantly increased by addition of *Cdx2* or *HNF1α*. There was no significant increase in *villin* expression in cells infected with the virus combinations tested (Supplementary Fig 4).

## 3 Discussion

Although BM itself does not arise spontaneously in rodents, there are several murine rodent-based models of the condition (reviewed in (Kapoor et al., 2015)). Moreover, mechanisms underlying gut differentiation are similar in all mammals and the requirements of tissue supply and *in vitro* culture make it necessary to use an animal model for experimental purposes. We have developed an adult explant culture model that recapitulates the full repertoire of cell types found in the oesophagus (basal, suprabasal and differentiated layers). The presence of a myofibroblast connective tissue layer beneath the basal cells allows for epithelial-mesenchymal interactions and might help to maintain the oesophageal phenotype in culture and account for the model's long-term viability. Above the connective tissue layer, the K14-positive basal cells differentiated and expressed the markers involucrin and K4, prior to the formation of the cornified cell envelope in fully mature squames.(Seery and Watt, 2000) Loricrin is a major component of the cornified cell envelope found in terminally differentiated squamous cells and has been demonstrated in epidermal keratinocyte cultures but to date has not been demonstrated in any *in vitro* oesophageal model (Hohl et al., 1991). Loricrin is located in the epithelial component of the outgrowth demonstrating that all stages of oesophageal squamous cell differentiation are represented. This model of squamous oesophagus allows for the assessment of the effects of ectopic gene expression on squamous differentiation in the context of columnar metaplasia.

We examined the role of calcium in the differentiation of SSQE. Calcium is an essential determinant of epidermal keratinocyte proliferation and differentiation (Hennings and Holbrook, 1983, Hennings et al., 1980). Mouse epidermal keratinocytes cultured in media containing less than 0.1 mM calcium do not stratify, proliferate rapidly and exhibit wide intercellular distances (Hennings and Holbrook, 1983, Hennings et al., 1980). Calcium at concentrations higher than 0.1 mM provoke an increase in stratification, terminal differentiation and cell-cell contacts (Hennings et al., 1980, Hennings et al., 1981). We found that oesophageal cells grown under low calcium conditions behave in a similar way with increased proliferation and lack of stratification and differentiation. Increasing the calcium concentration provokes the formation of cell-cell contacts and the appearance of differentiation markers such as K4.

Several lines of evidence have previously suggested that Cdx2 is implicated in the initiation of BM. We tested the ability of Cdx2 to induce the conversion of oesophageal cells to intestinal cells. However, adult oesophageal explant cultures fail to express any intestinal markers following Cdx2 infection despite the fact that we can obtain efficient Cdx2 expression in the K14-expressing cells in both normal and low calcium culture conditions. The only effect, apparent in the monolayer cultures, is a tendency for loss of p63 from *Cdx2*-expressing cells. Although at first sight a surprising result, it is consistent with other recent studies. Immortalized oesophageal cells require overexpression of the cell-cycle regulator cyclin D1 along with demethylating agents before ectopic *Cdx2* expression can provoke the expression of intestinal genes (Kong et al., 2009). Likewise, a transgenic study in which *Cdx2* is driven from the K14 promoter, demonstrated lack of intestinal gene expression in the oesophagus (Kong et al., 2011).

The transgenic experiments in which Cdx2 expression in the stomach provoked intestinal development involved initial upregulation of *Cdx2* at foetal stages (Silberg et al., 2002, Mutoh et al., 2002), and it has not been established whether *Cdx2* overexpression in the adult stomach has the same effect. One reason why *Cdx2* might provoke an intestinal phenotype in foetal but not adult stomach is because the proximal half of the rodent foetal stomach is lined by primitive columnar cells. This suggests that conversion to a columnar phenotype could be a prerequisite before induction of a differentiated intestinal phenotype. This consideration led us to examine HNF4α, which is expressed in stomach but not in oesophagus in early development (Duncan et al., 1994).

We show here for the first time a potential role for HNF4α in the development of the columnar phenotype in BM. When HNF4α is ectopically expressed in oesophageal cells there is a reduction in the number of cells expressing p63 and a robust induction of *villin* and, to a lesser extent, *Tff3*. The presence of *Tff3* is significant because it is considered as a marker of differentiated goblet cells (Velcich et al., 2002). In low calcium cultures HNF4α also induces E-cadherin expression. The induction of *E-cadherin* and *K8* is in keeping with the role of HNF4α in epithelialisation and tight junction formation. *HNF4α* can provoke epithelialisation of a dedifferentiated hepatoma cell line (H5) (Spath and Weiss, 1998). *HNF4α* null embryos lack *E-cadherin* expression, adherens junction proteins and exhibit large intracellular gaps (Battle et al., 2006, Parviz et al., 2003). The induction of *E-cadherin* in *HNF4α*-transduced cultures may also reflect functional regulation by the transcription factor. In the intestine, *E-cadherin* is expressed at a higher level in differentiated enterocytes in the villus region compared to the crypt (Escaffit et al., 2005).

Although *Tff3* was expressed, we did not observe expression of *Muc2* with *HNF4α*. However it is noteworthy that *Muc2* expression is minimally altered in *Hnf4α* null intestine (Garrison et al., 2006). The ability of *Hnf4α* to induce a partial intestinal phenotype in non-intestinal/non-hepatic cells has also been demonstrated in NIH-3T3 fibroblasts and MIA PaCa-2 pancreatic cell lines. Stable, retrovirally induced *Hnf4α* expression provoked the induction of *apolipoprotein A-IV* and *villin* in both cell lines as well as *Tff3* mRNA in fibroblasts (Babeu et al., 2009). The induction of *Tff3* and *villin* mRNA in oesophageal explants in the present study is in keeping with these findings.

While our results do not provide any particular evidence for a role of HNF1α in the formation of BM, the results presented here show for the first time that HNF4α induces a columnar phenotype with some intestinal features in oesophageal cells (K8, villin and Tff3). The question therefore arises whether *HNF4α* is also involved in the development of BM. The presence of HNF4α has previously been shown in BM (Piessen et al., 2007)**,** and we have confirmed this by immunostaining of our own human biopsies. Therefore we consider *HNF4α* induction a prime candidate as an early initiating event in the formation of BM. The results are consistent with the oesophageal epithelium being the cell of origin for BM although cannot exclude other possibilities such as oesophageal glands or multipotent cells left over from embryonic life (Barbera and Fitzgerald, 2010, Wang et al., 2011, Nicholson et al., 2012). Further investigation will be required to establish the cause of *HNF4α* induction, and whether the HNF4α protein is found in oesophagitis.

## 4 Materials and methods

All experiments were repeated at least three times.

### 4.1 Immunohistochemistry of human tissue

Formalin-fixed wax-embedded sections of archival biopsy forceps specimens of human oesophagus (normal and BM), gastro-oesophageal junction (GOJ), stomach, small intestine and colon were obtained from the Pathology unit at the Royal United Hospital Bath (REC number: 13/YH/0197). Immunohistochemical staining for CDX2 (1:80) and HNF4α (1:80) was carried out using a polymer detection system and DAB label. Briefly, for Cdx2, tissue sections were dewaxed, rehydrated, submerged in a low pH solution (BioGenex Antigen Retrieval Citra Plus Solution), microwaved until boiling and for two minutes thereafter. Sections were heated for a further 15 min in a 99 °C waterbath, allowed to cool for 20 min, transferred to PBS and sequentially treated with 3% peroxide block and BioGenex Power Block for 10 min each. Cdx2 antibody (BioGenex, Mouse) was diluted 1:80 (BioGenex Enhanced Antibody Diluent) and incubated with the sections for 30 min. Sections were rinsed thoroughly in PBS and treated with the Super Enhancer and Polymer-HRP reagent for 20 and 30 min respectively. Antibody detection was carried out in the presence of DAB (10 min). For HNF4α, sections were submerged in Dako EnVision™ Flex Target Retrieval Solution (high pH - diluted according to manufacturer's instructions), microwaved until boiling and microwave simmered for a further 20 min. Slides were allowed to cool for 20 min and transferred to PBS. Sections were rinsed in PBS and blocked for 2 h in 2% Roche blocking buffer followed by sequential treatments with the BioGenex peroxide and blocking solution as above. Hereafter, sections were subjected to the same protocol of antigen labelling and detection as for Cdx2 with HNF4α (Santa Cruz, Rabbit) also being diluted 1:80. Sections were counterstained with Gill's haematoxylin (Vector Laboratories).

### 4.2 Culture of adult squamous mouse oesophageal epithelium

All animal experiments were performed in accordance with UK Home Office regulations. Oesophagi were removed from adult CD1 mice following cervical dislocation and dissected in Minimum Essential Medium Eagle (MEM) with Hank's salts supplemented with 10% FBS (Invitrogen, Paisley, UK), penicillin/streptomycin (50U, Sigma) and 2 mM L-glutamine (all from Sigma-Aldrich, Poole, UK). The oesophagus was cut at the proximal and distal ends to ensure that gastric and buccal mucosa were excluded. It was opened longitudinally and the epithelium stripped from the underlying connective tissue. Each sample of oesophageal epithelium was dissected into approximate 1 mm^2^ sections and the samples from different individual mice were cultured separately. Sections (10−15) of epithelial tissue were then inserted into furrows that had been etched onto plastic coverslips. The coverslips were then placed in a 35 mm tissue culture dish and covered with 1.5 ml of Basal Medium Eagle (BME) with Earle's salts (Sigma-Aldrich, Poole, UK) supplemented with 20% foetal bovine serum, penicillin/streptomycin (50U, Sigma) and 2 mM L-glutamine.

For culturing oesophageal explants under low calcium conditions, MCDB 153 (Autogen Bioclear, Wiltshire, UK) medium was supplemented with L-glutamine (6 mM), human epidermal growth factor (5 ng/ml), ethanolamine (6.1 μg/ml), α-phosphoethanolamine (14.1 μg/ml), hydrocortisone (0.5 μg/ml) and bovine insulin (5 μg/ml) (all Autogen Bioclear).

### 4.3 Culture and generation of stable Het-1A cell line

Het-1A cells (ATCC, Middlesex, UK) were maintained in Basal Medium Eagle medium (BME) (Sigma) supplemented with 10% (v/v) foetal bovine serum (Gibco), 2 mM L-glutamine (Sigma) and penicillin/streptomycin (50U, Sigma). Culture medium was replaced every 2 days, and cells were subcultured (1:10) every 5–7 days.

Stable HNF4α expressing Het-1A cells were generated through lentiviral infection with pL-S-Hnf4α-I-EGFP28. Briefly, the lentivirus was prepared by transfecting pL-S-Hnf4α-I-EGFP and the packaging constructs pVSV-G, pREV, pGal/Pol/PRE into HEK293T cells (ECACC, Porton Down, U.K). Virus containing medium was harvested 48 h after transfection, diluted in complete BME medium supplemented with dextran (5 µg/ml) and added to 6.9×104 Het-1A cells for 24 h. Medium was changed every 2 days following infection. Cells were split and seeded onto 96-well plates for single cell colony selection with HNF4α expression being validated by immunofluorescence and RT-PCR.

### 4.4 Immunostaining of explant cultures and mouse oesophageal sections

Fixation and immunostaining of explant cultures or adult mouse oesophageal sections was performed as described previously (Yu et al., 2005). Primary antibodies were obtained and diluted as described in Table 1. Nuclei were stained with 0.1 µg/ml of 4′, 6-diamidino-2-phenylindole (DAPI). Images were either collected on a Leica DMRB fluorescent microscope with a digital camera or a Zeiss LSM 510 confocal microscope. We determined the specificity of antibodies directed against squamous epithelial epitopes by immunohistochemistry on adult mouse oesophageal sections (p63, K14, K4 and loricrin). In addition, we also determined the expression of villin in sections of adult mouse intestine and oesophagus. All oesophageal and intestinal proteins were expressed appropriately (Supplementary Fig 5).

### 4.5 Construction of Cdx2 and Cdx2-VP16 adenoviral vectors

Two viruses were constructed: one with the VP16 transactivation domain from *Herpes simplex* and one without. The VP16 virus was fused to the 5′ end of the full length mouse *Cdx2* cDNA (from Dr Debra Silberg University of Pennsylvania, USA). *Cdx2* was subcloned into a *VP16*-containing plasmid by *ClaI* digestion. The AdEasy expression system (Stratagene) was used for adenovirus delivery into cells and explant cultures. Briefly, *BglII* and *XhoI* were used for subcloning *Cdx2* and *VP16-Cdx2* into the pShuttle-IRES-hrGFP construct. The resulting shuttle vectors were then linearized with *PmeI* and cotransformed into BJ5183 electrocompetent cells with pAdEasy-1, the supercoiled viral DNA plasmid. Recombination was identified by restriction enzyme digestion analysis. The recombinant constructs were then produced in bulk in XL-10 Gold cells. Purified recombinant adenovirus plasmid DNA was digested with *PacI* to expose its inverted terminal repeat (ITR), and then used to transfect HEK239 cells where deleted viral assembly genes were complemented *in vivo*.

### 4.6 Expression of transgenes by adenoviral infection

Transgenes were expressed in cultured epithelium and Het-1A cells using first generation, replication defective, recombinant, adenoviral vectors: *Ad-null, Ad-RSV-GFP, Ad-CMV-Cdx2-hrGFP, Ad-CMV-VP16-Cdx2-hrGFP, Ad-CMV-HNF1α* and *Ad-CMV-HNF4α*.(Martinez-Jimenez et al., 2006).

Each explant culture was incubated with 5×10^7^ infectious units of adenoviral vector in 2 ml of complete BME for 12 h. Oesophageal explants grown under low calcium conditions were incubated with 5×10^5^ IU of adenovirus in 2 ml of MCDB 153 media (Autogen Bioclear, Wiltshire, UK) for 12 h. Explants were processed for RT-PCR or immunohistochemistry up to 7 days post-infection.

Het-1A cells were exposed to *Ad-null, Ad-CMV-HNF4α, Ad-CMV-VP16-Cdx2-IRES-hrGFP* and *Ad-HNF1α* alone or in combination (as indicated) to an MOI of 15 in the presence of dextran (5 µg/ml) for 24 h. Cells were harvested for analysis 4 days post infection.

### 4.7 Reverse transcription and polymerase chain reaction

RNA extraction, 1st strand cDNA synthesis and reverse transcription polymerase chain reaction was performed as described previously.(Li et al., 2007) Annealing temperatures and primer sequences are shown in Table 2. Quantitative real-time RT-PCR (qRT-PCR) was carried out using a LightCycler 1.5, Roche and reagent mix (FastStart SYBR Green Master, Roche). Primer sequences and annealing temperature are shown in Table 2.

## Conflict of interest

The authors disclose no conflict of interest.

## Author contributions

Study concept and design: **BC, JMWS, DT**. Acquisition of data: **BC, LG, YC, ZDB, WYY.** Analysis and interpretation of data: **BC, LG, ZDB, YC, JMWS, DT.** Drafting of the manuscript: **BC, LG, ZDB, JMWS, DT.** Critical revision of the manuscript for important intellectual content: **RJ, MB, JMQ, JMF, SGW, JMWS.** Material support: **WYY, RJ, MB, LB.** Study supervision: **JMF, JMWS, SGW, DT**.

## Funding

This work was funded by Cancer Research UK, Medical Research Council and the UK India Education and Research Initiative.

## Summary statement

To date the molecular mechanisms underlying Barrett's oesophagus remain unidentified. We provide evidence for a role of the transcription factor HNF4a in the switch from stratified squamous to columnar epithelium.

## Figures and Tables

**Table 1:** Primary antibodies used in immunohistochemistry.

Primary antibody	Manufacturer	Dilution	Species
anti-smooth muscle actin	Sigma-Aldrich, Poole, UK	1:100	Mouse
anti-cytokeratin 4	Sigma-Aldrich, Poole, UK	1:100	Mouse
anti-pan p63 (4A4)	Santa Cruz Biotechnology, California, USA	1:50	Mouse
anti-loricrin	Covance Princetown, USA	1:100	Rabbit
anti-cytokeratin14	Covance Princetown, USA	1:200	Rabbit
anti-cytokeratin 8/18	Developmental Studies Hybridoma Bank, University of Iowa, USA	1:200	Rat
anti-E-cadherin	BD Transduction Laboratories, New Jersey, USA	1:100	Mouse
anti-HNF4α	Santa Cruz Biotechnology, California, USA	1:100	Rabbit
anti-Cdx2	Biogenex, San Ramon, California, USA	1:100	Mouse

**Table 2:** Primers used for reverse transcriptase PCR.

Gene	Forward Primer	Reverse Primer	Annealing temp	Product size (bp)
HNF4α	GAAATGCTTCCGGGCTGGC	CTGCAGCTCCTGGAAGGGC	59	487
βActin	AAGAGCTATGAGCTGCCTGA	TACGGATGTCAACGTCACAC	54	160
βActin^a^	TAGGCACCAGGGTGTGATGG	CATGGCTGGGGTGTTGAAGG	58	323
ALPI	TGGATGCTGCCAAGAAGCTGC	AGAGATAGGCGGTTGCTGTGC	56	243
Cdx1	GA CGCCCTACGA ATGGATGC	CAGGTTAGCAGCCAGCTCG	58	184
Cdx2	CCATCACCCGCATCATCACCCG	AGTGAAACTCCTTCTCCAGCTCCAGC	60	272
Hnf4α	ACAGGAGAGGGTCAGAAGCA	GATGTTTGCACAACCACAGG	58	180
K14	GACTGGTACCAGAGGCAGCGGC	GGCATTGTCCACGGTGGCTGC	56	108
Lactase	TGCCCATCGACTGGAATGAGC	TGTCTCATGCTGCTGCTCGC	56	192
Muc2	GCAGTATCAGGCCTGTGGC	CACAATCTCGGTCTTCACTTCG	56	430
Muc5ac	GTGCAGGGCTCAGTTCTTTC	TGGTCTCTGTTTTCGTGCTG	56	224
Tff3	AGA TTA CGT TGG CCTGTC TCC	TCA GAT CAG CCT TGT GTTGGC	56	341
SI	GGC AAG ATC CTG TTT CCT GGA	CGA GCC TTA GGA ACA TAG CCA	56	271
Villin	TATGATATCCACTACTGGATTGGC	GCTTGAGTGCAGCCTTAGCG	54	586
Villin^a^	TTCCTGGCTTGGGATCCCTT	CCACTTTGGGGCTTGTGAC	68	121

a Denotes primers used for qRT-PCR.
