# Supplementary material for: Hnf4α is a key gene that can generate columnar metaplasia in oesophageal epithelium
Source: Differentiation. 2017 Jan-Feb;93:39–49. doi: 10.1016/j.diff.2016.11.001 (PMC5293356; doi:10.1016/j.diff.2016.11.001)
Supplement: Supplementary file 1 — Supplementary material [file mmc1.pdf]

Supplementary Figure 1

Immunofluorescent staining for K14 (green) in an adult oesophageal explant cultured in BME for 3 months. DAPI (blue) staining is also shown.

Supplementary Figure 2

Immunofluorescent staining for p63 (red) in oesophageal explants cultured in MCDB 153 and infected with Ad-CMV-Cdx2-hrGFP (A-C) or Ad-RSV-GFP (D-F). Ectopic Cdx2 expression is demonstrated by the GFP in (A and C). Arrows indicate Cdx2 infected cells that have lost p63 expression (A-C). Control infected cells remain positive for p63 (D-F). Scale bars represent 200 µm (A-C) and 100 µm (D-F).

Supplementary Figure 3

Immunofluorescent staining for p63 (green) and HNF4α (red) in oesophageal explants cultured in MCDB 153 and infected with Ad-Null (i-iii) or Ad-CMV-HNF4α (iv-vi). Ad-Null infected cells remain positive for p63 (ii). Scale bars represent 50µm.

Supplementary Figure 4

qRT-PCR analysis of villin expression in Het-1A cells (A) and the Het-1A-HNF4α c1 clone (B) infected with Ad-Null, Ad-Cdx2, Ad-HNF4α and Ad-HNF1α virus as indicated. Expression of villin was normalised to Ad-HNF4 infected cells or the Het-1A-HNF4α c1 clone. Data are shown as mean values ± standard deviation (S.D) (n=3).

Supplementary Figure 5

Immunohistochemical staining for p63 (A), K14 (B and C), Loricrin (D) and Villin (F) in adult mouse oesophagus; and Villin (E) in adult mouse intestine. Scale bars represent 100 μm

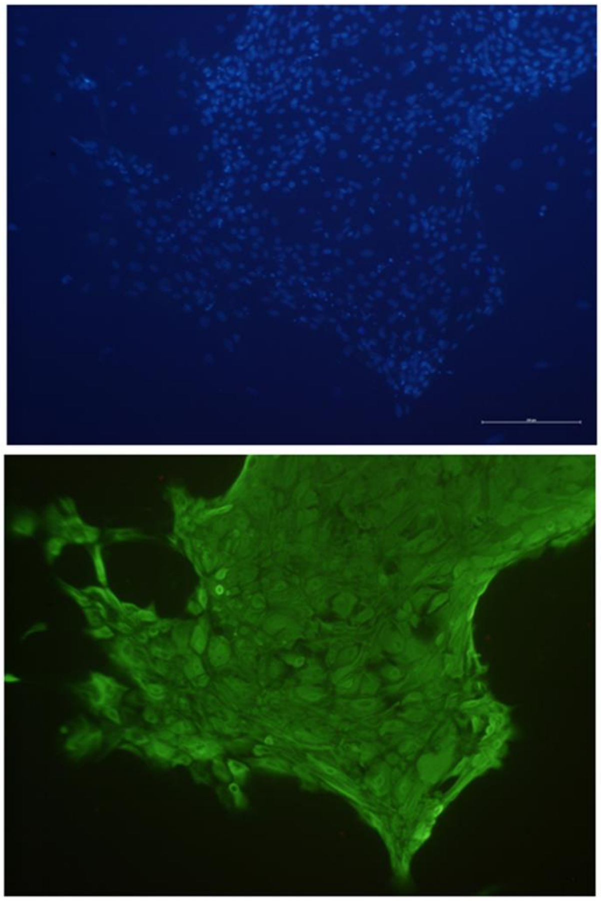

Supplementary Figure 1

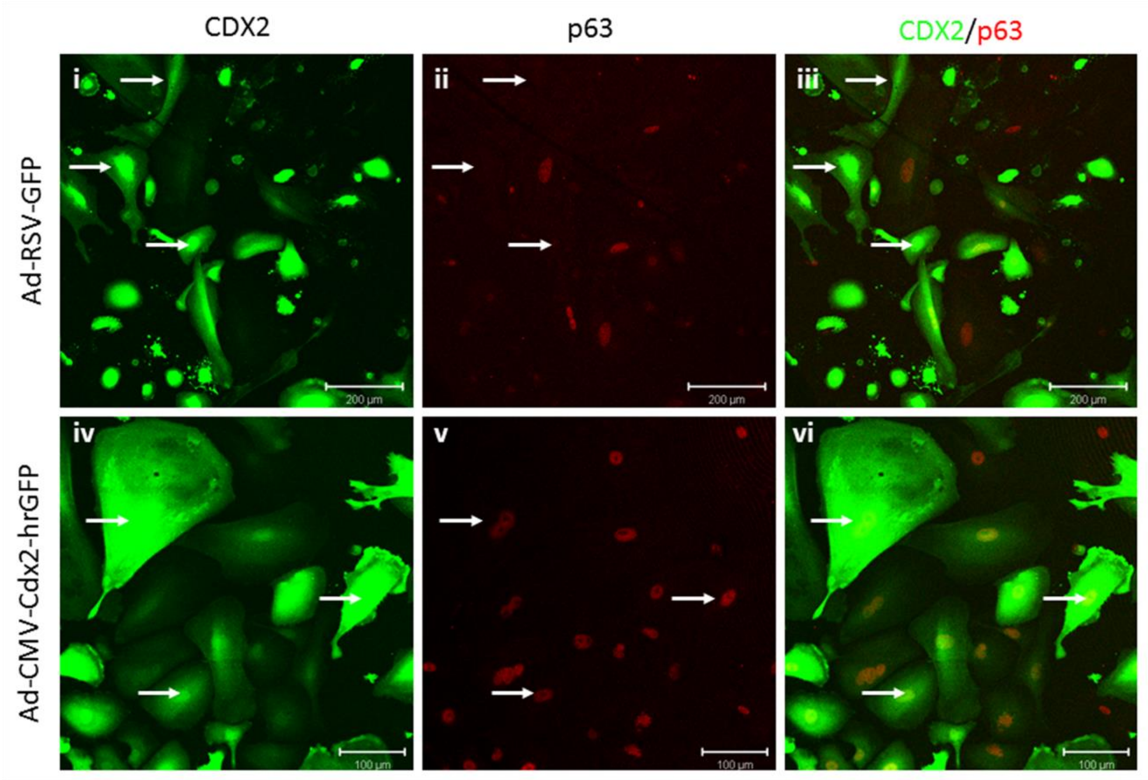

Supplementary Figure 2

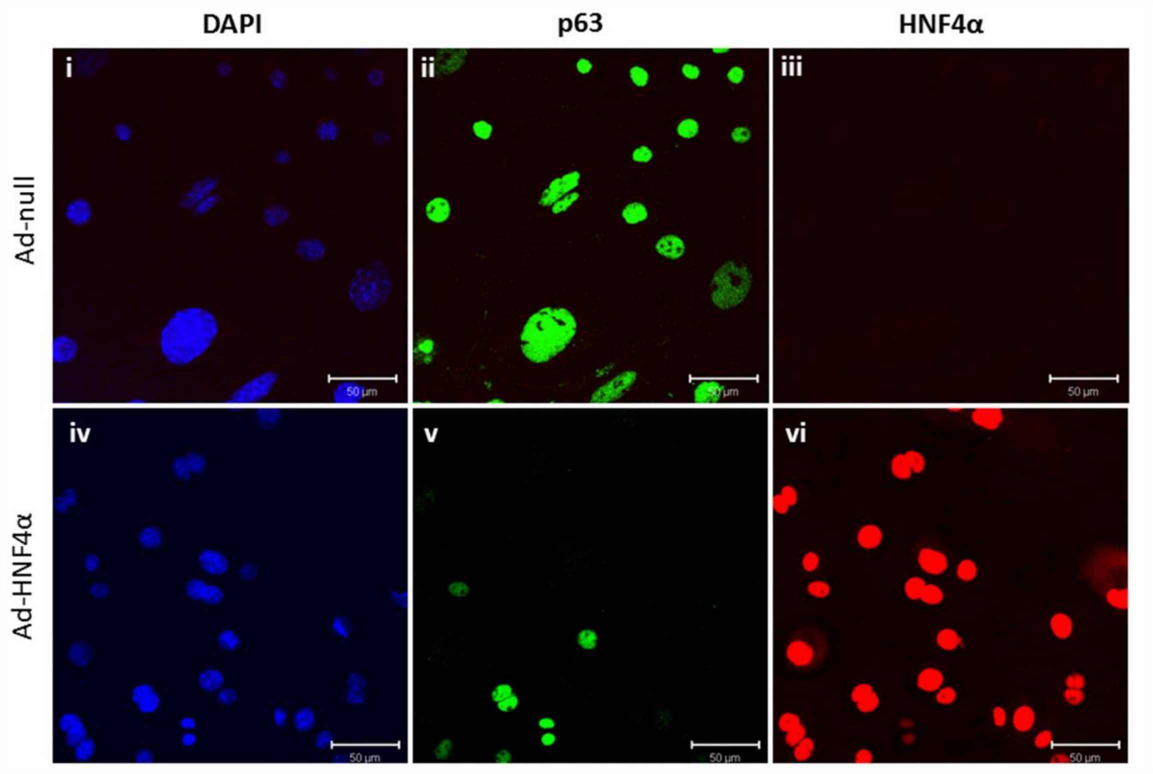

Supplementary Figure 3

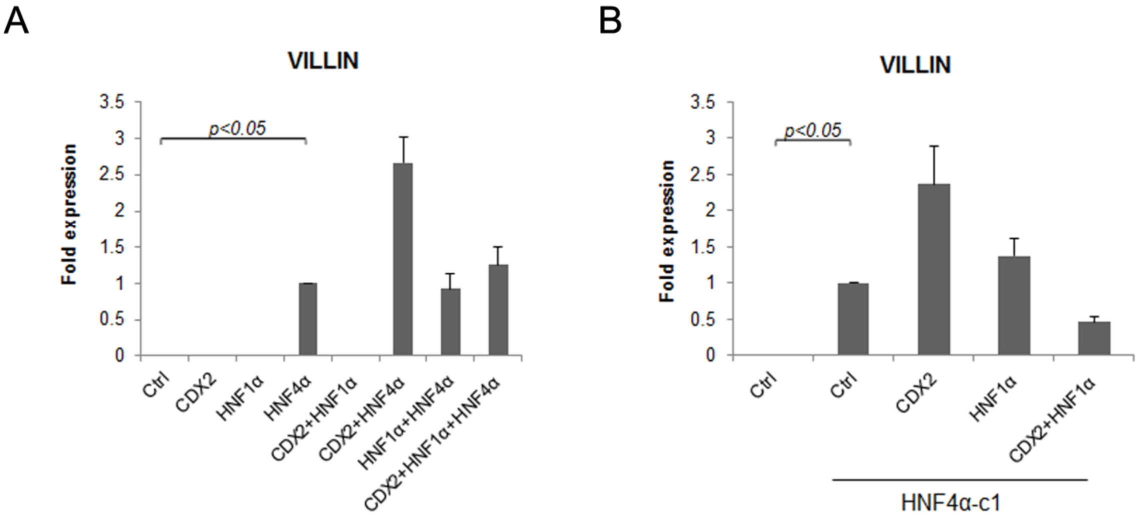

Supplementary Figure 4

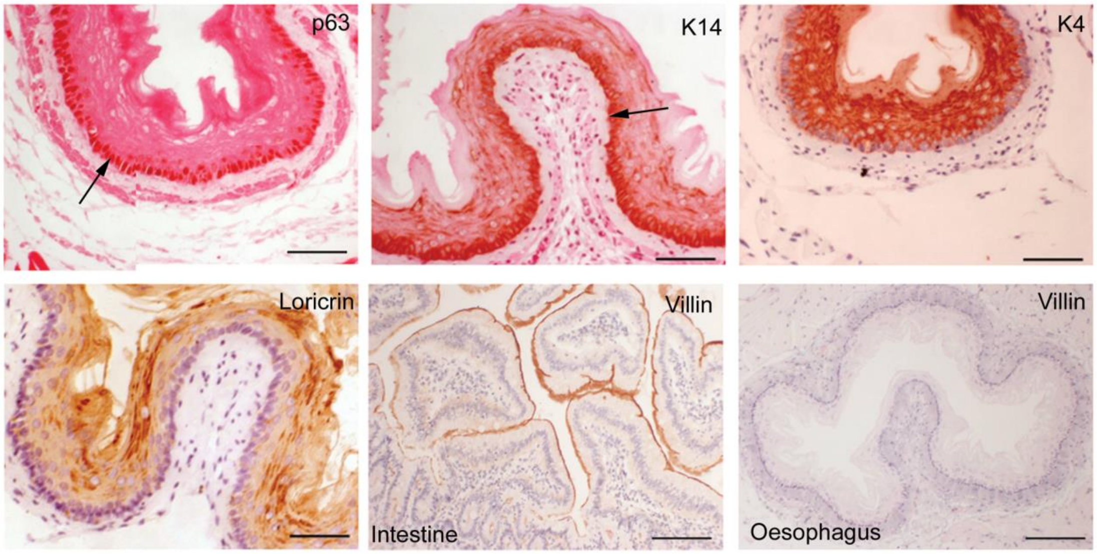

Supplementary Figure 5
